# Molecular Optomechanics Approach to Surface-Enhanced Raman Scattering

**DOI:** 10.1021/acs.accounts.1c00759

**Published:** 2022-07-01

**Authors:** Ruben Esteban, Jeremy J. Baumberg, Javier Aizpurua

**Affiliations:** † Materials Physics Center CSIC-UPV/EHU, Paseo Manuel de Lardizabal 5, 20018 Donostia-San Sebastián, Spain; ‡ Donostia International Physics Center DIPC, Paseo Manuel de Lardizabal 4, 20018 Donostia-San Sebastián, Spain; § NanoPhotonics Centre, Cavendish Laboratory, Department of Physics, University of Cambridge, Cambridge CB3 0HE, U.K.

## Abstract

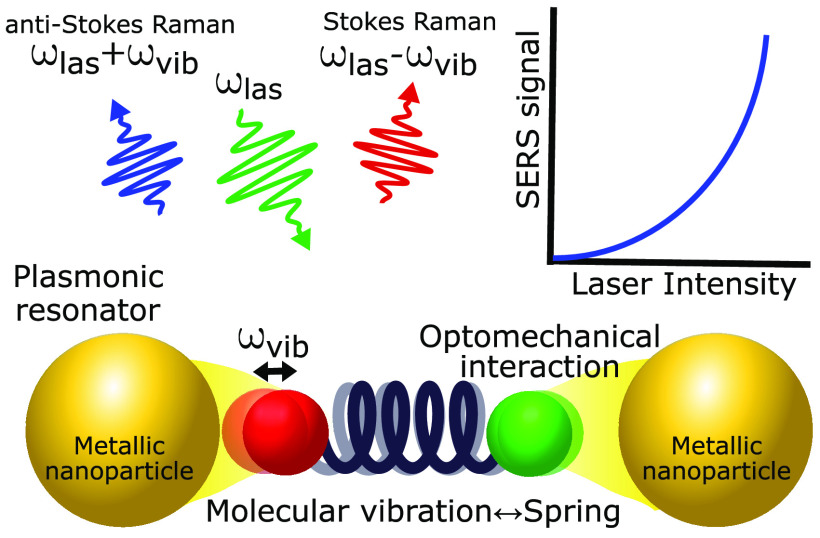

Molecular vibrations constitute one of the smallest mechanical oscillators available for micro-/nanoengineering. The energy and strength of molecular oscillations depend delicately on the attached specific functional groups as well as on the chemical and physical environments. By exploiting the inelastic interaction of molecules with optical photons, Raman scattering can access the information contained in molecular vibrations. However, the low efficiency of the Raman process typically allows only for characterizing large numbers of molecules. To circumvent this limitation, plasmonic resonances supported by metallic nanostructures and nanocavities can be used because they localize and enhance light at optical frequencies, enabling surface-enhanced Raman scattering (SERS), where the Raman signal is increased by many orders of magnitude. This enhancement enables few- or even single-molecule characterization. The coupling between a single molecular vibration and a plasmonic mode constitutes an example of an optomechanical interaction, analogous to that existing between cavity photons and mechanical vibrations. Optomechanical systems have been intensely studied because of their fundamental interest as well as their application in practical implementations of quantum technology and sensing. In this context, SERS brings cavity optomechanics down to the molecular scale and gives access to larger vibrational frequencies associated with molecular motion, offering new possibilities for novel optomechanical nanodevices.

The molecular optomechanics description of SERS is recent, and its implications have only started to be explored. In this Account, we describe the current understanding and progress of this new description of SERS, focusing on our own contributions to the field. We first show that the quantum description of molecular optomechanics is fully consistent with standard classical and semiclassical models often used to describe SERS. Furthermore, we note that the molecular optomechanics framework naturally accounts for a rich variety of nonlinear effects in the SERS signal with increasing laser intensity.

Furthermore, the molecular optomechanics framework provides a tool particularly suited to addressing novel effects of fundamental and practical interest in SERS, such as the emergence of collective phenomena involving many molecules or the modification of the effective losses and energy of the molecular vibrations due to the plasmon–vibration interaction. As compared to standard optomechanics, the plasmonic resonance often differs from a single Lorentzian mode and thus requires a more detailed description of its optical response. This quantum description of SERS also allows us to address the statistics of the Raman photons emitted, enabling the interpretation of two-color correlations of the emerging photons, with potential use in the generation of nonclassical states of light. Current SERS experimental implementations in organic molecules and two-dimensional layers suggest the interest in further exploring intense pulsed illumination, situations of strong coupling, resonant-SERS, and atomic-scale field confinement.

## Key References

- SchmidtM. K.; EstebanR.; González-TudelaA.; GiedkeG.; AizpuruaJ.Quantum Mechanical Description of Raman Scattering from Molecules in Plasmonic Cavities. ACS Nano2016, 10, 6291–629810.1021/acsnano.6b0248427203727.^1^*Introduction of a cavity-optomechanical framework to address SERS of molecules in plasmonic nanoantennas.*
- BenzF.; SchmidtM. K.; DreismannA.; ChikkaraddyR.; ZhangY.; DemetriadouA.; CarnegieC.; OhadiH.; de NijsB.; EstebanR.; AizpuruaJ.; BaumbergJ. J.Single-molecule optomechanics in “picocavities. Science2016, 354, 726–72910.1126/science.aah524327846600.^2^*Experimental proof of single-molecule optomechanical interactions by exploiting the extreme field localization of a plasmonic picocavity formed by single-atom protrusions.*
- ZhangY.; EstebanR.; BotoR. A.; UrbietaM.; ArrietaX.; ShanC.; LiS.; BaumbergJ. J.; AizpuruaJ.Addressing molecular optomechanical effects in nanocavity-enhanced Raman scattering beyond the single plasmonic mode. Nanoscale2021, 13, 1938–195410.1039/D0NR06649D33442716.^3^*Consideration of the full plasmonic response to correctly address the nanoscale optomechanical interaction which induces broadening and a spectral shift of the SERS signal.*
- SchmidtM. K.; EstebanR.; GiedkeG.; AizpuruaJ.; González-TudelaA.Frequency-resolved photon correlations in cavity optomechanics. Quantum Science and Technology2021, 6, 03400510.1088/2058-9565/abe569.^4^*Detailed theoretical analysis of the color correlations exhibited by scattered Raman photons.*

## 1 Introduction

Molecular vibrations contain rich information about the physical and chemical properties of molecules and their interaction with the environment. These vibrations can be addressed optically using Raman scattering, a powerful characterization technique in which photons of visible or near-infrared light (energy ℏω_las_ ≈ 1.5–2.5 eV, with ℏ being the reduced Planck constant) interchange energy with molecular vibrations occurring at much lower energy ℏω_vib_ ≈ 25–200 meV (≈ 200–1600 cm^–1^). The resulting inelastic emission of Stokes and anti-Stokes photons at slightly smaller ℏ(ω_las_ – ω_vib_) or larger ℏ(ω_las_ + ω_vib_) energies, respectively, leads to a characteristic fingerprint of peaks in the emission spectra. This fingerprint is mostly determined by the chemical bonds within the functional groups but is also sensitive to the temperature, the molecular surroundings, and the isotopic composition of the molecules, among other influences.

**Figure 1:**
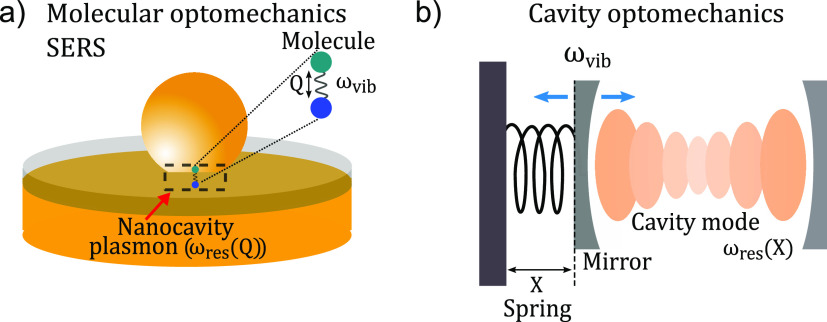
Comparison between molecular and conventional optomechanics. Sketch of (a) a SERS configuration consisting of a molecular vibration coupled to a plasmonic mode and (b) a canonical optomechanical system consisting of a Fabry–Pérot cavity where the motion of one of the mirrors induced by a vibrational mode varies the cavity length.

The weak Raman scattering cross section of individual molecules limits the performance of this spectroscopy technique. However, the signal is hugely boosted in surface-enhanced Raman scattering (SERS),^5,6^ where molecules are placed near metallic nanostructures acting as nanoresonators (Figure 1a). This makes it possible to characterize very small numbers of molecules or even a single molecule.^7,8^ The enhancing mechanism of SERS has been traditionally assigned to two main sources: first, a chemical enhancement^9^ produced by the bonding of the molecule to the metal, inducing repolarization and/or charge transfer, and second, an electromagnetic enhancement, usually the largest contribution, related to the intense plasmonic fields excited in metallic nanostructures, which very efficiently couple to molecular vibrations.

In this Account, we describe the recently developed quantum optomechanical description of (nonresonant) SERS that goes beyond this simple picture and explicitly considers the inelastic interaction between cavity plasmons and molecular vibrations at the origin of SERS (Figure 1a). This new approach establishes a connection between SERS configurations and standard optomechanical systems, where a mechanical oscillation of a cavity is coupled to one of its electromagnetic modes (Figure 1b).^1,10,11^

## 2 Classical Description of SERS

The electromagnetic field that interacts with the molecules in SERS configurations arises from the excitation of localized surface plasmon polaritons. Plasmonic resonances in metallic nanoresonators can strongly enhance the electromagnetic field at visible and near-infrared wavelengths λ and confine it to extremely small volumes,^12,13^ far below the ∼(λ/2)^3^ diffraction limit of free photons, leading to a huge increase in the SERS signal, as typically explained through the following classical picture.^5,14^ The local electric field exciting the molecule *E*(ω_las_) is enhanced by a factor *K*(ω_las_) = *E*(ω_las_)/*E*_0_, with *E*_0_ being the amplitude of the incident illumination (local intensity increased by |*K*(ω_las_)|^2^). This enhanced field induces a Raman dipole whose (frequency-shifted) emission rate is also strongly enhanced by the large local density of states (LDOS) of the plasmonic resonance.^13,15^ When the optical reciprocity theorem is applied, this increase in the emission rate can be related to the square of the field enhancement at the Raman emission frequency, |*K*(ω_las_ ± ω_vib_)|^2^. (See refs (14) and (16) for details.) The classical electromagnetic enhancement of the emitted SERS intensity EM_class_^SERS^ can thus be expressed as
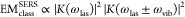
1

The total enhancement factor in eq 1 is often simplified to |*K*(ω_las_)|^4^, which is useful in estimating the order of magnitude of enhancement at the expense of accuracy: the energy of typical vibrations ℏω_vib_ can be comparable to the line width of plasmonic resonances, so *K*(ω_vib_) and *K*(ω_las_ ± ω_vib_) can be rather different. We note that the SERS signal scales with the fourth power of the field *enhancement*, i.e., the square of the intensity *enhancement*, but in this classical framework, it remains linear with the intensity of the excitation laser.

Equation 1 emphasizes that a key to maximizing SERS is to increase the field enhancement as much as possible. Plasmonic structures composed of two (or more) nanoparticles separated by nanometer gaps have proved to be particularly well suited for this purpose.^17,18^ A related configuration that has the advantage of being precise and relatively straightforward to integrate with molecular self-assembled monolayers (SAMs) or with 2D materials is the nanoparticle-on-mirror (NPoM) configuration, where a metallic nanoparticle is deposited on a metallic substrate with the SAM acting as a separator between them.^19^ Another important configuration which naturally produces a controllable gap is the junction between a tip and a substrate sandwiching molecules in-between, as in scanning probe microscopy such as tip-enhanced Raman spectroscopy (TERS).^20^

The simple enhancement estimate in eq 1 has proved to be very useful for the interpretation of many results in SERS, but it also shows limitations in the description of nonlinear effects or correlations, for instance. These limitations can be overcome with an optomechanical description, as described in this Account.

## 3 Optomechanical Description of SERS

### 3.1 Vibrational Population Dynamics

**Figure 2:**
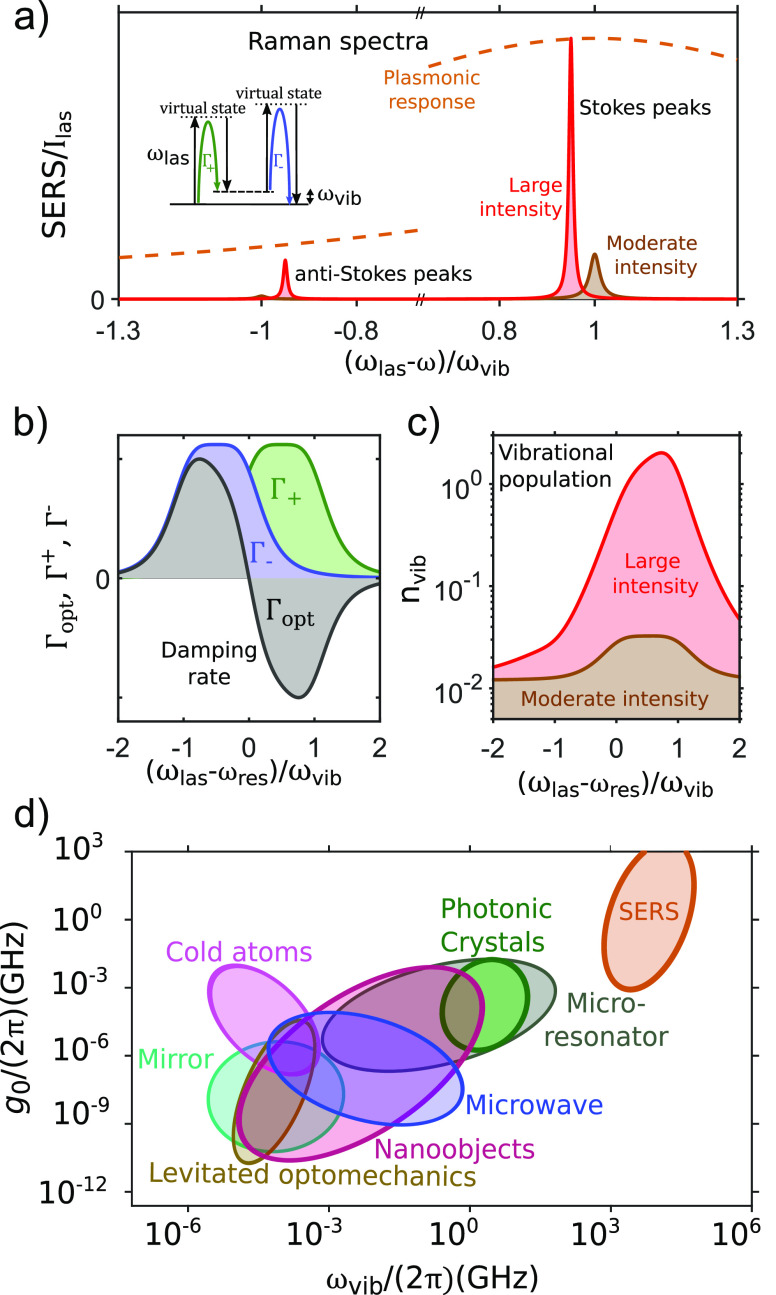
Principles of molecular optomechanics. (a) Sketch of characteristic SERS spectra of a molecule in a plasmonic resonator for larger (red curve) and lower (brown curve) illumination intensities. The brown dashed line indicates the plasmonic response. (b, c) Dependence on the incident laser frequency detuning of both (b) the optomechanical parameters Γ_+_ (green line), Γ_–_ (blue line), and Γ_opt_ (gray line) and (c) the vibrational population for large (red line) and moderate (brown line) illumination intensities in a single-mode plasmonic cavity with κ = ω_vib_. (d) Comparison of optomechanical coupling *g*_0_ and vibrational frequency ω_vib_ characterizing different types of optomechanical systems,^24^ including SERS configurations.

The emission of Raman photons at slightly decreased (*S*(ω_las_ – ω_vib_), Stokes line) or increased (*S*(ω_las_+ω_vib_), anti-Stokes line) energy is associated with an inelastic process that creates or annihilates molecular vibrations, respectively. *S*(ω_las_ – ω_vib_) and *S*(ω_las_ + ω_vib_) arise from the (incoherent) population of vibrations, *n*_b_, at ω_vib_, and the incident laser intensity, *I*_las_, as
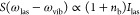
2
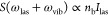
3Representative Raman spectra as a function of emission frequency for a single vibrational mode in the presence of a plasmonic resonator are sketched in Figure 2(a) for both the Stokes and the anti-Stokes lines (two incident intensities considered as discussed below). Under weak illumination intensity, the vibrational populations follow a thermal distribution, *n*_b_ = *n*_b_^th^ = [e^(ℏω_vib_/*k*_B_*T*)^ – 1]^−1^, where *k*_B_ is the Boltzmann constant and *T* is the temperature. In this regime, the ratio *S*(ω_las_+ ω_vib_)/*S*(ω_las_- ω_vib_) allows an estimation of the temperature of a medium, after correcting for the frequency-dependent response of the plasmonic resonator.^21^

By contrast, under large incident illumination intensity, the coupling of the molecular vibration and the cavity plasmon can optically pump the molecule, modifying *n*_b_. A *corrected* vibrational population can be obtained in the framework of cavity optomechanics when an appropriate plasmon-vibration coupling term (or optomechanical coupling) is included in the Hamiltonian describing the Raman process.^1,10,22^ The evolution of the vibrational population can then be obtained by solving the Langevin equations^10^ or the master equation^1^ for the corresponding density matrix, including incoherent optomechanically induced decay and pumping, in addition to the thermal pumping and the intrinsic vibrational losses γ_vib_. This approach is typically equivalent to solving a semiclassical rate equation of vibration creation and annihilation under the influence of optomechanical pumping and decay rates:^22,23^
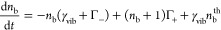
4Here, Γ_+_ and Γ_–_ are the incoherent optomechanical pumping and decay rates, respectively, which are introduced by considering the plasmonic resonator to be a reservoir affecting the vibrational dynamics. The optomechanical interaction destroys vibrations at a rate of Γ_*n*_b_ and creates them at rate of Γ_+_(1 + *n*_b_), with Γ_+_ and Γ_+_*n*_b_ corresponding to spontaneous and stimulated processes.^1^ For a single Lorentzian plasmonic mode of frequency ω_res_,^22^
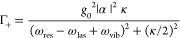
5
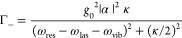
6where κ is the plasmonic decay rate, α is the amplitude of the plasmonic mode under laser illumination (|α|^2^ ∝ *I*_las_/[(ω_res_ – ω_las_)^2^ + κ^2^/4] for small and moderate laser intensity, *I*_las_), and *g*_0_ is the single-photon optomechanical coupling rate. This *g*_0_ is the key magnitude that determines the optomechanical interaction in SERS (section 3.2). Equations 4–6 are obtained under the assumption of optomechanical weak coupling, *g*_0_ ≪ κ, the typical situation in optomechanics, considering harmonic vibrations and neglecting an optomechanically induced shift of the plasmonic resonance. Γ_±_ contains both the enhanced excitation of the molecule and its enhanced emission, analogous to the classical SERS enhancement (eq 1).

The steady-state solution of the vibrational population is
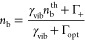
7where the optomechanical damping rate Γ_opt_ = Γ_–_ – Γ_+_ is defined.

According to eqs 5 and 6, the optomechanical rates Γ_+_ and Γ_–_ depend on the optical response at the Stokes, ω_las_ – ω_res_, and anti-Stokes, ω_las_ + ω_res_, frequencies, respectively. Depending on the detuning between the illumination and the plasmonic mode, both Γ_+_ > Γ_–_ and Γ_+_ < Γ_–_ are possible. This can be observed in Figure 2(b), which shows the explicit frequency dependence of Γ_+_ (green curve), Γ_–_ (blue curve), and the total optomechanical damping rate Γ_opt_ (gray curve) in the presence of a single plasmonic mode at frequency ω_res_. These three rates are proportional to the intensity of the incident laser *I*_las_ (eqs 5 and 6). Γ_opt_ is negative when the illumination frequency is blue-detuned from the mode (ω_las_>ω_res_) and positive when red-detuned.

The vibrational population also depends on the incident light intensity and frequency through Γ_+_ and Γ_–_ (eq 7), as observed in Figure 2(c). Moreover, the optomechanical pumping and annihilation of vibrations modify the line width of the Raman lines for sufficiently intense illumination, which can be understood as a change in the effective rate of molecular losses from γ_vib_ to γ_vib_ + Γ_opt_. The sign of Γ_opt_ becomes particularly important for large intensites where |Γ_opt_| ≈ γ_vib_. A narrowing of the line width (under blue-detuned illumination) with increasing intensity is illustrated in Figure 2(a), where the Stokes and anti-Stokes peaks in the Raman spectra are shown for moderate and strong laser intensities. The shift of the Raman line, also apparent in the figure, is discussed below.

### 3.2 Optomechanical Coupling at the Nanoscale

The optomechanical pumping and annihilation rates introduced in the previous section (eqs 5 and 6) depend on the optomechanical coupling, *g*_0_, which together with the losses and the frequency and intensity of the incident light determines the nonlinear regimes of SERS. The coupling *g*_0_ can be obtained by quantizing the plasmonic field and the molecular vibrations, following the standard procedure. The electric field operator **Ê** of a single plasmonic mode is mostly determined by the effective volume *V*_eff_ describing the localization of the electromagnetic field,^22,25^, with *â*^†^ and *â* being the plasmon creation and annihilation operators, ε_0_ being the vacuum permittivity, ε_d_ being the (linear and nondispersive) relative permittivity of the surrounding medium, and **u**(**r**) being a normalization function giving the spatial distribution and polarization of the plasmonic field. This field induces a nonresonant polarization of the electronic molecular transitions, given by the molecular polarizability . The polarizability is modified by the vibration of the molecule as , with as the zero-point fluctuation amplitude of the vibration,  as the Raman tensor of vibrational mode *k*, and *b̂*^†^ and *b̂* as the creation and annihilation operators associated with the generalized coordinate Q _*k*_. Q _*k*_ parametrizes the vibration (assumed to be perfectly harmonic) and depends on the properties of the molecule, including its mass.^6^ In the following text, we consider just a single vibrational mode and write . Hence we express the induced Raman dipole operator , whose interaction with the plasmonic mode leads to the optomechanical interaction Hamiltonian
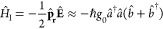
8where *g*_0_ is the (single-photon) optomechanical coupling strength, given by
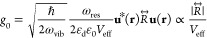
9The additional Hamiltonian terms describing the plasmonic and vibrational excitations as well as the laser excitation of the system follow the standard form. The prefactor 1/2 is included in eq 8 because **p̂**_**r**_ is an induced dipole moment operator.^13^ A detailed discussion of this framework can be found in ref (22).

Equation 8 has been derived to model SERS but is formally identical to the interaction Hamiltonian that describes traditional optomechanical systems^24^ such as a Fabry–Pérot optical cavity where one of the mirrors supports mechanical vibrational modes. The connection between these two previously unrelated disciplines is further emphasized by an alternative interpretation of the interaction Hamiltonian *Ĥ*_I_.^10,11^ By adding *Ĥ*_I_ to the Hamiltonian *Ĥ*_res_ = ℏω_res_*â*^†^*â* describing the cavity mode at energy ℏω_res_, we obtain *Ĥ*_I_ + *Ĥ*_res_ = ℏ(ω_res_ - *g*_0_(*b̂* + *b̂*^†^))*â*^†^*â*. Thus, the excitation of the vibrational modes induces a shift, *g*_0_(⟨*b̂*⟩ + ⟨*b̂*^†^⟩), of the cavity mode frequency (⟨ ⟩ indicates the expectation value). For example, in a Fabry–Pérot configuration, the mechanical oscillation of a macroscopic mirror changes the length of the optical cavity, modifying the resonant energy of the optical mode^24^ (Figure 1(b)). A similar mechanism arises in SERS (Figure 1(a)): the microscopic vibration of the atoms modifies the polarizability of the molecule by , which shifts the energy of the plasmonic mode. (In a simple picture, this shift is due to the electromagnetic coupling of two strongly detuned dipoles, with one representing the plasmonic mode and the other representing the molecular excitation.)

Although their interaction Hamiltonians are analogous, SERS and traditional optomechanical systems explore a different set of energies, losses, and coupling rates. Plasmonic modes exhibit very large losses (small quality factors), but the vibrational frequencies ω_vib_ and the optomechanical coupling strengths *g*_0_ are very large. The large ω_vib_ gives very low vibrational populations at room temperature, as desired for quantum applications. The huge value of *g*_0_ is a direct consequence of the plasmonic nanocavity confinement of electromagnetic fields to extremely small regions (very small *V*_eff_).^19,26,27^ As an example, Figure 2(d) indicates that the very large values of *g*_0_/(2π) and of the vibrational frequency ω_vib_/(2π) in typical SERS situations, as compared to other cavity optomechanical systems, greatly extend the optomechanical landscape.

## 4 Nonlinearities in SERS

**Figure 3:**
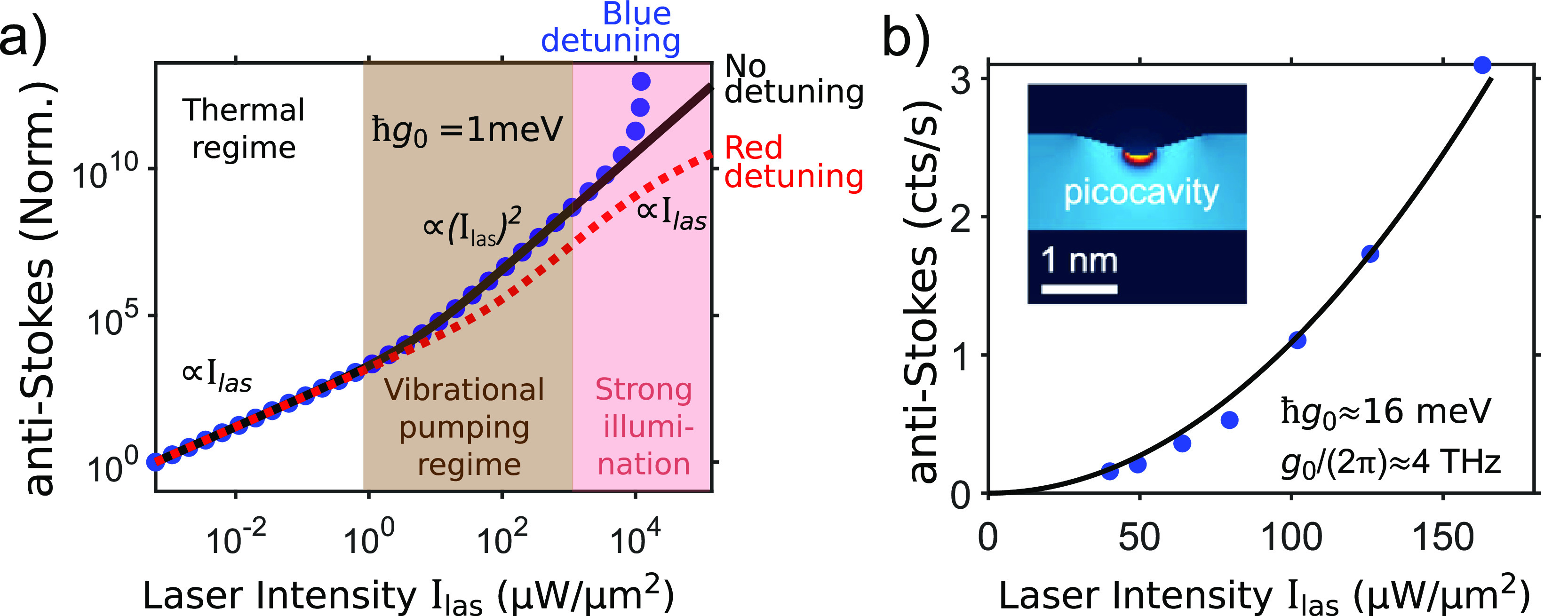
Nonlinear scaling in molecular optomechanics. (a) Scaling of the anti-Stokes signal vs laser intensity when the illumination is resonant (solid black line), blue-detuned (blue dots), or red-detuned (dashed red line) with respect to a single plasmonic mode. Results are normalized to values at the weakest intensity considered and are obtained with ℏ*g*_0_ = 1 meV, ℏω_res_=1.72 eV, ℏκ = 200 meV, ℏω_vib_ = 196.5 meV, ℏγ_vib_ = 0.07 meV, *n*_b_^th^ = 4 × 10^–4^, and ω_las_ – ω_res_ = 0, +ω_vib_, -ω_vib_, for no detuning, blue detuning, and red detuning, respectively. White-, brown-, and red-shaded areas correspond respectively to the thermal regime, vibrational pumping regime, and strong-*I*_las_ regime where parametric instability and saturation of the vibrational population can occur. (b) Measured evolution with laser intensity of the anti-Stokes signal in a NPoM configuration (symbols) and quadratic fitting (∝ *I*_las_^2^, solid line). The inset shows the simulated electric-field localization near a picocavity formed by an atomic-size protrusion. Adapted with permission from ref (2). Copyright 2016 American Association for the Advancement of Science.

We analyze in this section the consequences of optomechanical coupling in the evolution of the SERS signal as a function of incident illumination intensity, *I*_las_. Optomechanical pumping and decay can lead to three different regimes of SERS as *I*_las_ increases:

- Weak illumination (thermal regime): In most experiments, the laser intensity is sufficiently weak so that Γ_+_ and Γ_–_ are small and the vibrational population *n*_b_ is given by the thermal pumping *n*_b_ = *n*_b_^th^. The Stokes *S*(ω_las_ – ω_vib_) and anti-Stokes *S*(ω_las_ + ω_vib_) scattering rates are then proportional to the laser intensity (eqs 2 and 3 and Figure 3(a), white-shaded area). In this regime, the molecular optomechanics treatment is equivalent to the classical description,^22^ recovering the classical dependence in eq 1.
- Intermediate illumination intensity (vibrational pumping regime): For larger laser intensity, the system enters the vibrational pumping regime, where vibrations are predominantly created by the Stokes processes induced by pumping (Γ_+_) rather than by thermal processes so that the vibrational population *n*_b_ in eq 7 becomes proportional to the laser intensity *n*_b_ ∝ *I*_las_ (in this regime, Γ_+_ ≳ γ_vib_*n*_b_^th^ and Γ_opt_ ≪ γ_vib_). Importantly, the emission of an anti-Stokes photon requires the destruction of a vibration, so the anti-Stokes scattering is proportional to both *I*_las_ and *n*_b_ (eq 3) and thus scales quadratically with laser intensity (brown-shaded area, Figure 3a). The Stokes signal also acquires a quadratic contribution due to vibrationally stimulated Raman scattering^1^ (eq 2), but experimental observation of this effect is demanding because it requires a large vibrational population (*n*_b_ ≳ 1). These results are fully consistent with semiclassical models of vibrational pumping^22,28^ and have also been discussed in studies of Stokes–antiStokes correlations.^29^ An analysis of the quadratic anti-Stokes signal dependence allows an estimation of the optomechanical coupling strength *g*_0_, as has been demonstrated for the NPoM constructs^2^ [Figure 3(b)]. Combining the ultranarrow gap between the gold particle and substrate together with atomic-scale protrusions (picocavities) formed by the induced movement of gold atoms strongly localizes the field, which can address vibrations of individual molecules efficiently. Estimated values of *g*_0_ reached tens of meV, much larger than for any conventional cavity optomechanical system. Attempts to probe into the nonlinear regime of the Stokes signal use pulsed illumination,^30,31^ which better mitigates the damage to molecules while allowing large vibrational populations before bond breaking.
- Strong illumination (population saturation and parametric instability): For very large intensities (red-shaded area in Figure 3a), the modification of the total vibrational loss from γ_vib_ to γ_vib_ + Γ_–_ – Γ_+_ = γ_vib_ + Γ_opt_ (e.g., the denominator in eq 7) is critical. If Γ_opt_ is negative, then the effective losses become zero for a sufficiently intense laser, leading to the narrowing of the Raman line (Figure 2(a), red spectra) and to a divergence known as the parametric instability. In the context of SERS, these instabilities were first predicted using rate equations.^22,23^ In contrast, for Γ_opt_ > 0 the effective losses γ_vib_ + Γ_opt_ are increased, the Raman line broadens, the vibrational population eventually saturates (similarly to cooling in conventional optomechanical systems^24^), and the scaling of the anti-Stokes signal with laser intensity turns out to be linear again.^32^
  For a situation with a single plasmonic mode, the optomechanical instability and line-width narrowing occur when the laser energy is larger than the energy of the plasmonic resonance (blue detuning), whereas red-detuned illumination leads to vibrational saturation and broadening^24^ (red-dashed line, Figure 3a). We discuss in section 6 how this simple criterion is no longer appropriate for complex plasmonic systems. Nevertheless, the regime of very strong illumination offers the possibility to reveal intriguing new phenomena in SERS.
  We discuss in the Outlook the challenge of distinguishing optomechanical effects from other additional effects that can be present in SERS experiments. We also discuss the convenience of using pulsed illumination to obtain information on the excitations of the system in the time domain and to attain the large laser intensities required for inducing the system into a regime of substantial optomechanical coupling, without damaging the samples.

## 5 Collective Effects

In the last few decades, various SERS experiments have shown the ability to measure Raman scattering from single molecules,^7,8^ but very often target samples are constituted by molecular assemblies. In NPoM configurations, for instance, hundreds to thousands of self-assembled molecules can be placed in each NPoM gap.^19^ In typical theoretical calculations, these molecules are considered separately, adding the Raman scattering from each molecule as if they did not interact. This simple approach is valid under weak illumination conditions (thermal regime), but an extension of the optomechanical model^10,32^ to consider multiple molecules reveals that the single-molecule description fails at large laser intensity *I*_las_. In such a situation, the molecule–molecule correlations established via their mutual Raman interaction through the plasmonic resonator become large enough to modify the emitted Raman signal, revealing the collective nature of the response^33^ of the full molecular assembly.

**Figure 4:**
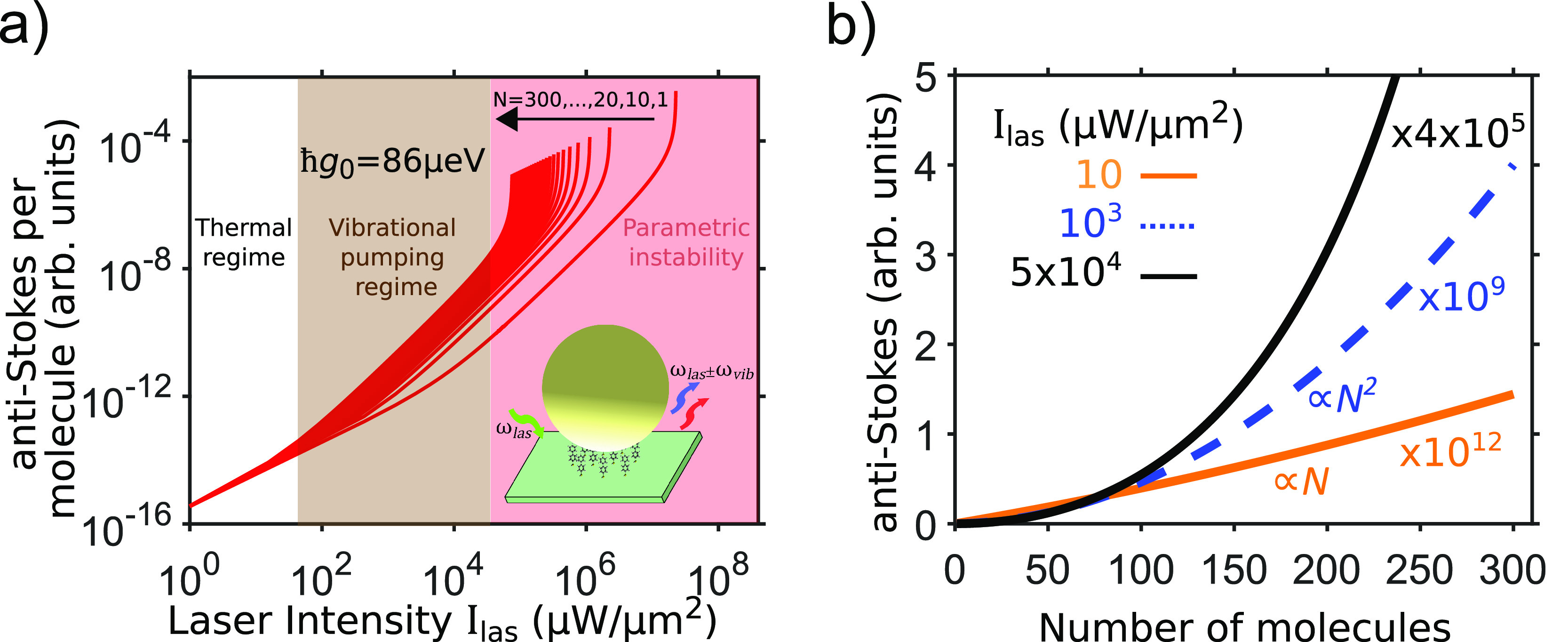
Collective effects for blue-detuned external illumination with respect to a single plasmonic mode. (a) Dependence of the anti-Stokes signal on laser intensity, normalized by the number of identical molecules *N*, plotted for different *N*. White-, brown-, and red-shaded areas correspond respectively to the thermal regime, vibrational pumping regime, and strong-*I*_las_ regime where parametric instability occurs. (b) Dependence of the total anti-Stokes signal on the number of molecules *N*, plotted for laser intensities *I*_las_ indicated. The values of *I*_las_ are chosen to show linear (orange line), quadratic (blue line), and faster-than-quadratic (black line) scaling with *N*. These scalings are characteristic of the thermal regime, vibrational pumping regime, and strong-*I*_las_ regime near the parametric instability. Adapted with permission from ref (32). Copyright 2020 American Chemical Society.

An increase in the number of molecules leads to a reduction of laser intensity thresholds needed to observe the nonlinear optomechanical effects at intermediate and strong illumination (section 4). For instance, the scaling of the anti-Stokes emission with *I*_las_^2^ induced by vibrational pumping (brown-shaded area in Figure 4a), the parametric instability (red-shaded area in Figure 4a), and the changes in the Raman line width all occur at lower intensities with more coupled molecules.

An analysis of the anti-Stokes signal as a function of the number of molecules *N* (Figure 4b) indicates a quadratic *N*^2^ scaling in the vibrational pumping regime (blue line), resembling superradiance.^34^ This superradiant-like scaling can be observed for weaker incident intensity if the temperature is reduced, making experimental observation easier. A more pronounced scaling with *N* is also attainable at large *I*_las_ (at a suitable laser frequency), i.e., when the system approaches the parametric instability (black line in Figure 4b).

The superradiant-like scaling and the change in the intensity thresholds can be understood by invoking the excitation of a collective vibrational bright mode that couples efficiently with the plasmon,^10^ similar to collective excitonic bright modes.^34,35^ For a simple situation with identical molecules, the optomechanical coupling strength associated with this bright mode scales with the square root of the number of molecules. Alternatively, these collective effects can also be understood as emerging from the correlations established between the different molecules, which synchronize their response.^32^

The collective response is often ignored in the interpretation of SERS, but it may be at the origin of unexpectedly large experimental signals.^36^ Recent work on the analysis of nonlinearities in the Stokes signal^30^ could reconcile the measurements and the predictions from molecular optomechanics only when the excitation of a collective bright mode involving ∼200 molecules was considered.

## 6 Complexity of the Plasmonic Resonator

**Figure 5:**
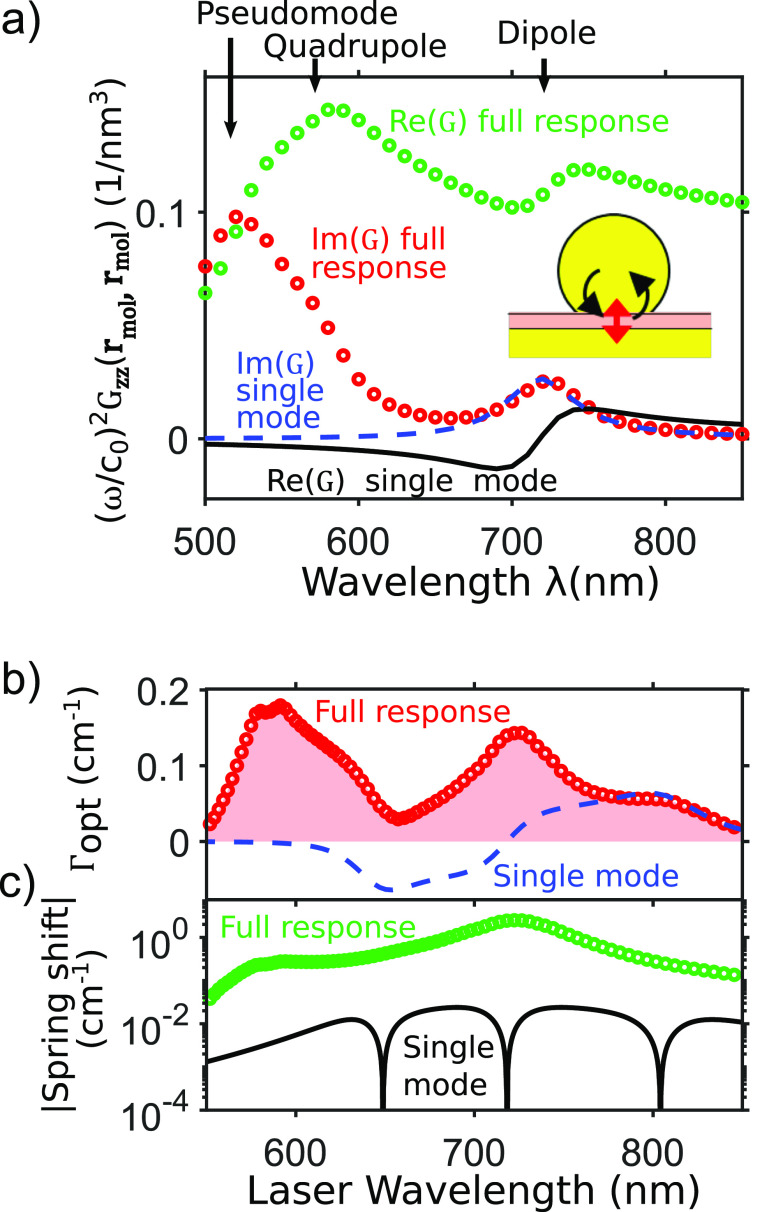
Effect of the full plasmonic response on molecular optomechanics. (a) Spectral dependence of the real (black line, green dots) and imaginary (blue line, red dots) parts of the self-interaction dyadic Green’s function *G*_*zz*_(**r**_mol_, **r**_mol_) at wavelength λ (frequency ω), obtained for a nanoparticle-on-mirror configuration with a dipole oriented along *z* (inset). (b) Optomechanical damping rate Γ_opt_ and (c) absolute value of the spring shift obtained for the same cavity as in (a) and *I*_las_ = 1*W*/*μm*^2^. Γ_opt_ and the spring shift are proportional to *I*_las_. In all panels, the solid black or dashed blue line corresponds to the simplified single-cavity-mode description, and red or green dots (and shaded red curve) correspond to the full continuum-field model. Adapted with permission from ref (3). Copyright 2021 the authors. Published by the Royal Society of Chemistry under a Creative Commons Attribution Noncommercial 3.0 Unported License.

Typical models in traditional cavity optomechanics consider the optical response of a cavity dominated by a single Lorentzian mode, a questionable approximation for a complex plasmonic nanocavity where several overlapping modes emerge. A more general description of the linearized optomechanical interaction was now developed that incorporates the full plasmonic response of an arbitrary nanosystem using a continuum-field formalism.^37^ The plasmonic response is described in such case via the field enhancement and dyadic Green’s function *G⃡* of the plasmonic resonator. The application of this continuum-field model to a NPoM nanocavity^3^ that exhibits a complex optical response (Figure 5a) clearly emphasizes the limitations of the single-cavity-mode approximation. Contributions from high-energy plasmonic modes (often described as a pseudomode^38^) that are fully included in the continuum-field description can invalidate the single-mode approximation, particularly because these high-energy modes strongly influence the dyadic Green’s function that describes the self-interaction of the molecule (Figure 5a). In Figure 5b, the spectral dependence of the optomechanical damping rate, Γ_opt_, for a single mode (dashed blue line) is compared with that obtained for the full plasmonic response (red curve), showing substantial differences. Contrary to the single-cavity-mode approximation (section 4), the continuum-field model indicates that the laser detuning (from the main mode) is not the only parameter determining the sign of Γ_opt_ and thus whether the Raman peaks narrow or broaden for large illumination intensities as well as the specific scaling of these peaks with laser intensity. Furthermore, the specifics of the optomechanical interaction also depend on the energy of the molecular vibration.

The optomechanical interaction can also strongly modify the energy ℏω_vib_ of the molecular vibrations, leading to a frequency shift of the Raman emission lines. This so-called optical spring shift^24^ is proportional to the laser intensity and is already present for a single plasmonic mode. However, including the full plasmonic response indicates that the excitation of mirror charges in the metallic surfaces can enhance this shift by 2 orders of magnitude, compared to considering a single mode^3,39^ [Figure 5(c)]. This facilitates the practical implementation of large vibrational energy shifts in molecules at reasonable laser intensities, thus opening a new avenue for optical control of chemical reactivity.

The optomechanical response can be particularly rich when a nanostructure with a complex plasmonic response interacts with many molecules (section 5). In this situation, different collective modes contribute to the scattered signal, with each mode experiencing a different spring shift and broadening (or narrowing) of the Raman line. Recent experiments^39^ with intense pulsed illumination have obtained unexpected scaling of the Raman signal with laser intensity *I*_las_, an effect attributed to the redistribution of the intensity of the emitted light between these collective modes. The emergence of a strongly shifted and strongly broadened bright collective mode can effectively saturate the intensity of the sharp Raman lines and can provide anomalous increases in the “background” signal. The complex plasmonic response and collective effects can thus have large impacts on practical implementations of molecular optomechanics at the nanoscale.

## 7 Statistics of SERS Emission

**Figure 6:**
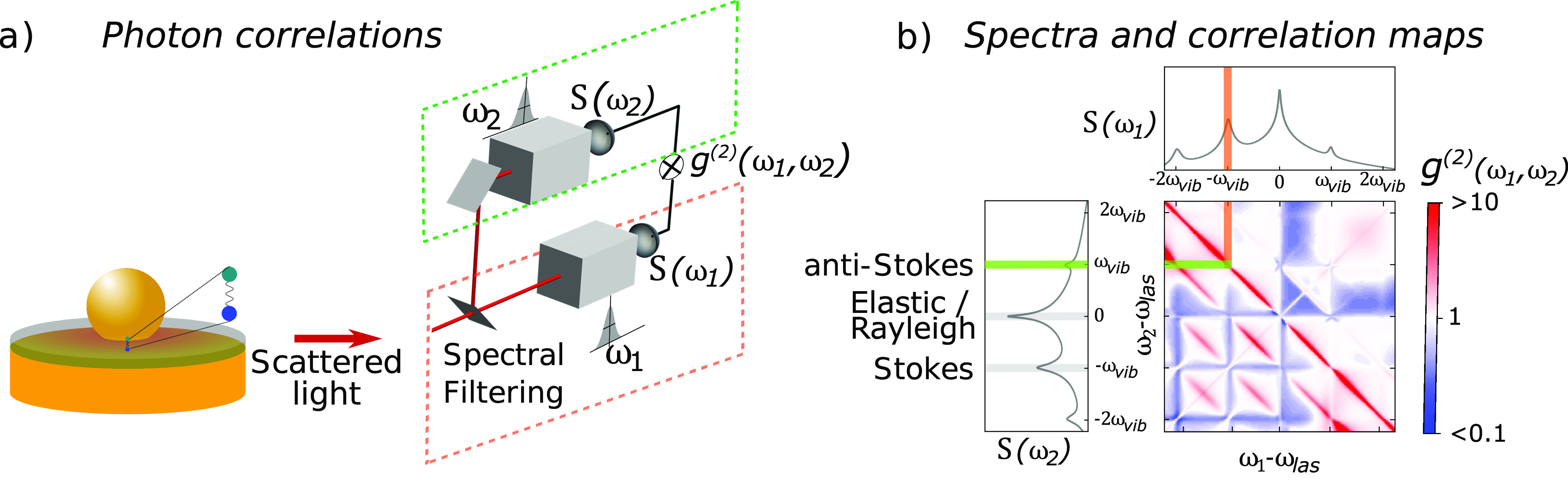
Correlations in molecular optomechanics. (a) Scheme of a typical setup for measuring two-photon correlations. (b) Example of the emitted Raman spectra (top and left) and 2D correlation map as a function of the frequency of the emitted photons. The correlation map is obtained for a single molecule and continuous-wave illumination. Reproduced with permission from ref (4). Copyright 2021 the authors. Published by IOP Publishing under the terms of the Creative Commons Attribution 4.0 License.

Typical SERS experiments obtain spectral information from the intensity of the scattered radiation. However, the emitted light also contains information on the photon statistics, which can be accessed, for instance, by measuring the second-order correlation between photons arriving at two detectors in a Hanbury Brown-Twiss configuration. Furthermore, interest in spectrally filtering the light before detection (Figure 6a) has recently been emphasized to extract the correlations of pairs of photons of different colors.^40^ For example, in Raman spectroscopy^29,41,42^ and more generally in molecular optomechanics,^1^ the correlations of Stokes and anti-Stokes photons can reveal very large photon bunching. Indeed, each anti-Stokes photon gains energy from a quantum of molecular vibration that (at zero temperature) has been created by the emission of a Stokes photon so that the possibility of these two photons arriving at the detector at the same time is typically much larger than the product of the individual probabilities.

The generalization of these results to the analysis of the second-order correlations between all pair of frequencies^4^ leads to two-dimensional correlations maps (Figure 6b) that reflect the complexity of the underlying emission processes. In addition to the Stokes/anti-Stokes bunching, the maps exhibit additional features associated with the intrinsic nonlinearity of the molecular optomechanics Hamiltonian, with two-photon leapfrog processes involving emission via a virtual state and with interference effects between Raman and elastically scattered photons. Information about the dynamics of these processes can be obtained by measuring the correlations as a function of the time delay between the two photons. Finally, the Cauchy–Schwarz inequality reveals that nonclassical states of light can be obtained.^41^ Thus, the measurement of photon correlations constitutes a powerful tool for characterizing SERS experiments and also for obtaining, for example, heralded phonons.^43^

## 8 Outlook

Molecular optomechanics describes SERS processes under very general conditions and predicts a variety of intriguing phenomena such as strong nonlinearities of the emitted signal, collective effects, nonclassical correlations, and heat transfer^44^ as well as narrowing, broadening, and energy shifts of the Raman lines. While experiments have begun to explore these effects,^2,30,39^ they are often challenging because the unambiguous identification of molecular optomechanical dynamics usually requires intense light fields that can damage the molecules and/or the plasmonic structures. To maximize the optomechanical coupling strength and thus minimize these issues, one can take advantage of strong field localization in plasmonic nanostructures. Plasmonic modes of nanocavities such as those in NPoM constructs and in STM configurations^20,45,46^ are characterized by very small effective volumes, on the order of 10–100 nm^3^, and can reach even smaller values with the emergence of picocavities^2,26,27,47^ within them.

Other alternatives include exploiting DNA origami to place individual molecules at well-defined positions in nanocavities^48,49^ or implementing nanolenses to increase the detected signal as well as the coupling to incoming lasers.^50^ Alternative approaches rely on the design of hybrid dielectric–plasmonic structures, which exhibit modes characterized by much smaller losses than in plasmonic resonances.^51,52^ In addition to the engineering of the optical response, an improvement of the chemical enhancement due to the binding of the molecules to metallic surfaces^9^ can also increase the optomechanical interaction. The use of some of these strategies to optimize the optomechanical coupling strength could lead to values comparable to plasmonic losses and thus to accessing interesting interaction regimes such as single-photon optomechanical strong coupling,^24,52^*g*_0_ ≈ κ. Molecular optomechanics also allows for exploring complex situations beyond standard optomechanics involving the rich multiresonant response of plasmonic cavities as well as simultaneous coupling with many molecules.

The physical (and chemical) mechanisms unearthed in SERS signals are challenging to interpret because the complexity of organic molecule–metal interactions and the rich dynamics governing plasmonic and molecular decay can affect the emitted signal in many different ways. Nonlinear scaling of SERS signals and broadenings of Raman lines with laser intensity can have other origins besides optomechanical interactions, such as structural molecular changes during irradiation, heating, decay from excitonic states, or the decay of plasmons into hot carriers.^28,53−55^ However, careful modeling of each experiment, together with suitable control measurements, can reveal the role of molecular optomechanics.^30,39^ For this purpose, it is also useful to repeatedly cycle the laser intensity to ensure that the sample is not damaged,^39^ to measure the transient Raman signal at very short time scales to characterize the system dynamics,^2,47^ and to calibrate the input and output energy carefully to obtain absolute values of Raman cross sections. Future possibilities to identify unambiguous optomechanical molecular dynamics might examine the correlations of emitted photons or, alternatively, access specific optomechanical signatures such as optomechanically induced transparency^24^ or a strong dependence on laser detuning.^1,22,32^ Coupling the plasmon with phonons in solids or van der Waals materials^31,56^ opens an alternative path to exploiting optomechanical interactions at the nanoscale.

In this context, the use of ultrafast pulsed illumination^30,54,57^ is particularly promising because it allows for stronger (peak) laser intensities without harming samples and for directly accessing the dynamics of vibrations by controlling the delay between pulses. Pulsed illumination can also be useful to study the influence of intramolecular vibrational redistribution (IVR)^54,58^ (the interchange of energy between different vibrations) and environment effects on molecular vibrational states and thus on Raman signals. Such studies use picosecond pulses or two-dimensional femtosecond spectroscopies.^59,60^ Moreover, time-resolved measurements of molecular dynamics can help to identify if the width of the Raman lines is due to either pure dephasing or to other decay processes.

Additionally, the optomechanical framework can be further generalized in the future to other situations of interest in SERS. For example, the emission of Raman photons from systems where vibrations are strongly coupled with infrared cavity modes^61−63^ is still poorly understood, and the optomechanical framework introduced here can address this situation. Furthermore, plasmonic structures can create regions of strong fields (hot spots) smaller than ∼1 nm^3^ via atomically sharp features that form picocavities.^26,64^ These atomic-scale hot spots enable us to address individual molecular vibrations,^2^ including those that are inaccessible to standard Raman spectroscopy, and even map them with submolecular resolution.^20,65,66^ A combined classical and quantum treatment can explain many of the results observed in these picocavities,^66,67^ but a full description of the optomechanical interaction in such a situation requires further elaboration. Moreover, the study of nonresonant SERS, as described in this Account, can be completed with the inclusion of electronically resonant SERS processes, driving a new variety of nonlinear effects.^68−70^

Advances in molecular optomechanics can also be useful in the design of new devices. It has recently been proposed that the sensitivity of the Raman signal to the molecular vibrational state can be exploited to fabricate terahertz detectors^71^ using a similar principle as in surface-enhanced sum frequency generation (SE-SFG^57^). These devices can exhibit a fast response and a low level of noise as compared to current technologies. The first experimental demonstrations of IR detection have recently been achieved.^72,73^ In conclusion, molecular optomechanics brings the field of optomechanics into the realm of the nanoscale with organic molecules and solid-state phonons, introducing a new plethora of physical and chemical effects in SERS and thus opening a new technological avenue for molecular nanotechnologies.
